# Innovative machine learning approaches for indoor air temperature forecasting in smart infrastructure

**DOI:** 10.1038/s41598-024-85026-3

**Published:** 2025-01-02

**Authors:** Nataliya Shakhovska, Lesia Mochurad, Rosana Caro, Sotirios Argyroudis

**Affiliations:** 1 Artificial Intelligence Department, Lviv Polytechnic National University, 12 S. Bandery St, Lviv, 79013 Ukraine; 2 Brunel University of London, Uxbridge, UB8 3PH UK; 3 Polytechnic University of Madrid, Madrid, 28040 Spain

**Keywords:** Surrogate modeling, Time series forecasting, LSTM, Energy efficiency, Smart buildings, Cumulative error analysis, Machine learning, Environmental sciences, Environmental social sciences

## Abstract

Efficient energy management and maintaining an optimal indoor climate in buildings are critical tasks in today’s world. This paper presents an innovative approach to surrogate modeling for predicting indoor air temperature (IAT) in buildings, leveraging advanced machine learning techniques. At the core of this study is the application of Long Short-Term Memory (LSTM) networks for time-series modeling, which significantly enhances the capture of temporal dependencies in temperature predictions. The proposed LSTM with RWCV (Rolling Window Cross-Validation) offers significant advantages over a usual LSTM in time-series tasks, particularly due to its ability to adapt to new data trends through the rolling window mechanism. It provides more robust and generalizable forecasts in dynamic environments, prevents overfitting through dropout and cross-validation, and improves model evaluation with temporal integrity. In contrast, traditional LSTM models are better suited for static, non-evolving datasets and may not handle dynamic time-series data effectively. To rigorously assess model performance, a comprehensive evaluation framework is developed, incorporating metrics such as mean square error (MSE) and the coefficient of determination (R²). Additionally, a novel cumulative error analysis method is introduced enabling real-time monitoring and model adjustment to maintain predictive accuracy over time. Test results demonstrate that model losses on the test dataset are only marginally higher than those on the training dataset, indicating robust generalization capabilities. Loss values range from 0.0004709 to 0.02819861, depending on building operating conditions. A comparative analysis reveals that Adaboost and Gradient Boosting models outperform linear regression, highlighting their potential for achieving energy-efficient and comfortable indoor climate management in buildings. The findings underscore the efficacy of the proposed approach for IAT prediction and point towards further research possibilities in dataset expansion and model optimization to enhance building climate management and energy conservation.

## Introduction

Maintaining a stable and comfortable indoor climate in buildings is essential for the health and well-being of occupants while also playing a critical role in optimizing energy consumption and reducing environmental impact. This is particularly significant in the context of rising energy prices and the pressing need to combat global warming. As a result, the construction industry is shifting toward “smart” buildings, which leverage automation to achieve maximum energy efficiency and occupant comfort. Additionally, new regulations and standards worldwide demand improved energy efficiency and indoor environmental quality in modern buildings^1–3^.

In this scenario, surrogate modeling has emerged as a transformative tool for enhancing building energy management and climate control. Surrogate models (SMs), or metamodels, are simplified representations of complex systems that approximate the behavior of detailed models at a fraction of the computational cost. These models enable faster forecasting and real-time decision-making, crucial for smart building applications. By integrating flexibility and adaptability, SMs ensure relevance and accuracy in dynamic environments while reducing the computational burden, making them essential components of modern building management systems^4^.

Surrogate modeling techniques have wide-ranging applications in engineering, simulation, and machine learning. For instance, they support optimizing heating, ventilation, and air conditioning (HVAC) systems by predicting indoor air temperature (IAT) based on environmental data. Accurate IAT predictions reduce energy costs, improve comfort, and lower greenhouse gas emissions. This creates a dual benefit of enhancing occupant well-being and mitigating climate change impacts.

Several types of surrogate models (SMs) exist, each with distinct characteristics and applications. These include polynomial regression, Gaussian processes, radial basis functions (RBFs), support vector machines (SVMs), among others. Polynomial regression uses polynomial functions to model dependencies between variables, being simple and straightforward, but less accurate for very complex functions^5^; Gaussian Process provides flexible modeling of complex dependencies using probabilistic approaches to estimate forecast uncertainty^6^; RBFs use distance-to-center functions to build smooth interpolation models, which is useful for problems with irregular data^7^; SVMs use hyperplanes to separate data in high-dimensional spaces where nonlinear kernels can model complex relationships^8,9^.

Thus, surrogate modeling is a powerful tool for solving complex, multidimensional problems where direct computation is impractical. By approximating complex models with simpler ones, it enables more efficient and effective analysis, optimisation, and decision-making.

The primary objective of this study is to develop and evaluate an efficient surrogate modeling framework for Indoor Air Temperature (IAT) prediction in smart buildings. This involves leveraging advanced machine learning techniques, optimized data sampling algorithms, and innovative evaluation strategies to address challenges in energy-efficient climate management.

This paper focuses on developing an efficient LSTM-based surrogate modeling approach to predict indoor air temperatures, emphasizing reduced computational costs, improved accuracy, and adaptability to evolving data patterns. By incorporating Rolling Window Cross-Validation (RWCV) and optimized data sampling algorithms, this study introduces a robust framework for addressing non-stationary datasets and enabling real-time applications.

The main results of this work are presented below:

- A novel approach to surrogate modeling for indoor air temperature prediction by integrating advanced machine learning techniques with optimized data sampling methods. While the use of LSTM networks for time series modeling is established, this study extends their application by combining them with Rolling Window Cross Validation. Explicitly enhances the capture of temporal dependencies by gradually increasing the training set size as new data points are added. This enables the model to learn patterns from a growing dataset, improving its forecasting accuracy for future time steps. The rolling window approach ensures that the model adapts to evolving trends over time, rather than relying on static historical data. This is particularly beneficial for datasets with non-stationary patterns where trends change over time.
- A methodology has been developed to reduce computational costs in creating SMs by applying optimized data sampling algorithms, such as latent hypercubic sampling and factorial design.
- Development cost efficient and compact LSTM model (50,851 parameters occupying approximately 203.4 kilobytes) is suitable for deployment in resource-constrained environments, ensuring practicality alongside high performance.

The rest of the paper is organized as follows: Sect. 2 provides a detailed overview of the current state of research in this area. Section 3 describes the dataset used, discusses the context of the paper, and presents the proposed method for predicting IAT in buildings using environmental and air quality indicators. Section 4 presents the results of numerical experiments and a comprehensive discussion of the proposed approach. Finally, Sect. 5 summarizes the main conclusions and offers recommendations for future research directions.

## Related works

Predicting IAT has emerged as a critical research focus, with traditional methods categorized into three major approaches:

1. White box models rely on physical principles and detailed building dynamics. These models are highly accurate but computationally intensive and difficult to adapt to real-time applications.
2. Gray box models combine physical insights with empirical data. While more computationally efficient, they may lack precision in complex environments.
3. Black box models employ data-driven machine learning (ML) techniques to make predictions without explicit physical modeling. These models offer scalability and adaptability, particularly for dynamic and non-stationary datasets, but often struggle with generalization across diverse contexts.

Studies like those by Villano, Mauro, and Pedace^10^ have extensively reviewed ML and deep learning (DL) methods for building energy management, emphasizing their potential and limitations. While CNN-LSTM models, as proposed by Elmaz et al.^11^, have demonstrated high accuracy (R² > 0.9) in IAT prediction, their architectural complexity necessitates substantial computational resources, making them less practical for resource-constrained settings.

However, the integration of convolutional layers with LSTM networks inherently increases the architectural complexity of the model, resulting in a larger number of trainable parameters. This complexity leads to higher computational demands during training and inference. Consequently, the CNN-LSTM approach necessitates substantial computational resources, such as GPUs or TPUs, and may require extended training times, particularly for large datasets.

Two strategies were developed in Mtibaa et al.^12^: LSTM-MISO for multi-input single-output and LSTM-MIMO for multi-output multi-output prediction. The performance of these strategies was evaluated based on two real smart buildings with variable (VAV) and constant (CAV) air volume systems. The experimental results showed a significant advantage of the LSTM model over multilayer perceptron models. In particular, it reduced the prediction error by 50%. The main drawback of this work is the limited focus on one machine-learning method, which may not provide the best results in all cases.

Hamayat et al.^13^, in turn, propose the use of a certain modification of Bi-LSTM, which is a black box model based on artificial intelligence and data-driven approaches. The results of the numerical experiments showed an improvement in IAT prediction of up to 10% using the proposed model compared to the standard LSTM model for IAT prediction.

The study by Liang et al.^14^ employs a surrogate modeling approach similar to ours, to replace complex physical models with simplified yet accurate data-driven models. However, their work has limitations, including the lack of consideration for time dependencies due to the use of the K-nearest neighbors (KNN) algorithm, the specificity of their model to a single region (Shanghai, China), and the restriction of input parameters to only seven key building characteristics. Additionally, their focus is limited to thermal loads, they rely on a single algorithm for modeling, and their evaluation of model performance is insufficiently comprehensive. Compared to our approach, which uses LSTMs to account for time dependencies, a wider set of parameters, multiple machine learning models, and comprehensive performance evaluation, the work by Liang et al.^14^ has limitations in terms of accuracy and versatility of predictions.

Zouloumis et al.^15^ present a model for predicting the required thermal capacity of buildings using multilinear regression and analyzing heat loss, heat capacity, and air infiltration. While this traditional approach demonstrates satisfactory accuracy, it has notable limitations. It does not account for complex time dependencies, making it less effective for handling large and dynamic datasets. In addition, lacks methods for reducing computational costs or analyzing accumulated error, which limits its applicability in real-time and increases computational costs. The lack of a comprehensive approach to evaluating model performance can also lead to a less objective assessment of the model’s effectiveness.

Langtry et al.^16^ examined the use of data to improve the accuracy of building condition forecasting models and the effectiveness of a model predictive control (MPC) scheme in a distributed generation and storage power system. Their findings revealed that a simple linear multilayer perceptron model could achieve forecast accuracy comparable to more advanced machine learning models, with the added benefits of requiring less data and computation. Using over two years of training data did not significantly improve the prediction accuracy for most of the models tested, indicating that collecting data over a long period is not essential for developing effective MPC schemes. In addition, change point analysis showed that removing low-similarity data and using online learning can improve data efficiency and forecasting accuracy. However, the application of reusing linear models to predict load between buildings can lead to increased prediction errors. These results indicate that despite the use of different prediction models and methods, there is a challenge in modeling the error between simulated and real building performance. While some machine learning models can provide equivalent prediction accuracy to most sophisticated models, they can be more sensitive to data and require significant computational resources. It has also been found that long-term data collection does not always lead to a significant improvement in prediction accuracy, which demonstrates the difficulty of accurately modeling real-world building performance based on available datasets. Thus, despite successes in leveraging data for predicting building performance, challenges such as error modeling persist, necessitating further research and the development of improved methodologies.

Various time series forecasting methods can be used to predict building efficiency and address the problem of modeling the error between simulated and real performance. For example, Kontopoulou et al.^17^, conducted a comparative analysis of ARIMA models alongside machine learning methods such as neural networks, support vector machines, decision trees, linear models, and deep learning. The effectiveness of using ARIMA and SARIMA models in forecasting is described by Petropoulos et al.^18^. Additionaly, the use of the Holt-Winters method^19^ accounts for trends and seasonality in time series, making it effective for predicting building performance when clear cyclic patterns are present. Each of the discussed methods has its advantages and limitations, and they can be used individually or in combination to obtain more accurate and reliable building performance forecasts. Given the complexity of error modeling, integrating different methods and continuously improving them can significantly reduce discrepancies between predicted and actual building performance.

Based on our literature review, we aim to solve the problem of accurately predicting the IAT in buildings while taking into account time dependencies and optimizing computational efficiency. Current research highlights various modeling approaches, ranging from white-box to black-box, each with distinct advantages and limitations. White-box models provide high accuracy but are complex and data-intensive, while black-box models, such as our proposed approach, prioritize simplicity and speed but may lose accuracy due to the lack of physical constraints. Our approach aims to use machine learning techniques and optimized data sampling algorithms to improve both prediction accuracy and computational efficiency in IAT modeling. By bridging the gap between accuracy and practicality, our goal is to develop a robust solution that is suitable for real-world applications, effectively addressing the trade-offs inherent in existing modelling frameworks. Key insights from literature analysis are given below:

1. Existing studies often focus on single methods or static datasets, limiting their ability to generalize across diverse building environments.
2. Many models lack the capacity to handle evolving trends in real-time, emphasizing the value of LSTM networks for temporal pattern recognition.
3. While traditional methods such as linear regression are computationally light, they fall short in capturing complex relationships, necessitating hybrid approaches like LSTM with RWCV.
4. The absence of robust mechanisms for cumulative error analysis hampers real-time application, a gap addressed by the current study.

Research gap details and needs are given in Table 1.

**Table 1:** Research gaps in indoor air temperature (IAT) prediction.

Limited generalization across diverse environments	Models are often trained on localized datasets and fail to generalize to different building types, climates, or locations (e.g., Liang et al., 2023).	Develop a model adaptable to a wide range of environmental and operational conditions.
Challenges in capturing temporal dependencies	Traditional surrogate models, like KNN or regression methods, fail to account for time dependencies in dynamic building environments.	Leverage advanced time-series models like LSTMs to handle temporal patterns and evolving trends.
High computational complexity	Advanced models, such as CNN-LSTMs or Transformers, are resource-intensive, limiting applicability in resource-constrained settings (Elmaz et al., 2021).	Design lightweight, memory-efficient models that maintain high accuracy for real-time applications.
Inadequate adaptation to non-stationary data	Static models require frequent retraining to adapt to new data trends, reducing efficiency in dynamic environments.	Incorporate mechanisms like Rolling Window Cross-Validation (RWCV) to enable dynamic adaptability.
Seasonal and floor-level variations	Models inadequately account for temperature differences due to seasonal changes or building floor levels.	Test models across multiple seasons and building configurations for consistent performance in varied conditions.
Balancing accuracy and efficiency	Many models sacrifice computational efficiency for higher accuracy, making them impractical for real-world use.	Develop models that strike an optimal balance between predictive accuracy and computational demands.
Error accumulation in long-term forecasting	Current models fail to address cumulative errors, leading to decreased accuracy in long-term predictions (Langtry et al., 2024).	Introduce cumulative error analysis to enhance the accuracy and reliability of long-term forecasts.

## Materials and methods

### Dataset description

The dataset used in this paper^20^, is related to the environmental conditions in the building and includes seasonal indicators, measurements from different floors, time stamps, CO2 concentration, measured and modeled IATs, and error calculations. This type of dataset is useful for analyzing the performance of simulation models compared to actual measurements, especially in the context of building energy management or indoor climate control systems.

The dataset contains several columns, each representing different features or measurements. Below is a detailed description of each column:

- season_w: This binary column indicates the presence or absence of winter season conditions. It might be a binary column where ‘1’ represents winter and ‘0’ represents non-winter.
- season_s: This binary column indicates the presence or absence of summer season conditions.
- floor2: This column represents data or measurements taken from the second floor of a building.
- floor1: This column represents data or measurements taken from the first floor of a building.
- floor0: This column represents data or measurements taken from the ground floor (or floor 0) of a building.
- House name: type of building. Two buildings were analyzed.
- Date: This column records the date of the measurement or observation. The format is a standard date format (e.g., YYYY-MM-DD).
- Time: This column records the time of the measurement or observation. The format is standard time format (e.g., HH: MM).
- Measured CO2: This column contains the measured concentration of CO2 (carbon dioxide) at the given date and time.
- Measured IAT: This column contains the measured indoor air temperature (IAT) at the given date and time.
- Simulated IAT: This column contains the simulated indoor air temperature (IAT) at the given date and time, generated by a simulation model.
- err: This column represents the error between the measured IAT and the simulated IAT. It is calculated as the difference between the two values.
- cumulative: This column contains the cumulative error over time, which is the running total of the absolute error.

Dataset consists of 16,067 instanses with 0 null values.

### Research pipeline

We used R version 4.1.2 to conduct this research, run on Spark 3.0.0, 8 cores.

1) Data preparation:

- Сumulative error calculation.

Cumulative error is the accumulated error that occurs over several stages of a process. This concept is relevant in numerical analysis, computer modeling, optimization, and data analysis^21^. Understanding and managing cumulative error is critical to ensuring the accuracy and reliability of results.

In the context of surrogate modeling, cumulative error encompasses all errors that occur during the construction and use of the model. Surrogate models are simplified versions of more complex and resource-intensive models, so managing cumulative error is essential to ensure their effectiveness and reliability.

To calculate the cumulative error in the context of surrogate modeling, use (1). Based on this formula, the total error accumulated during the entire modeling process can be calculated.

1 $$\:{E}_{c}\left(t\right)=\sum\:_{i=1}^{t}\left|E\left(i\right)\right|,$$

where $\:{E}_{c}\left(t\right)$ – is the cumulative error at time $\:t$, $\:\left|E\left(i\right)\right|$ – is the absolute value of the error at the $\:i$-th step, $\:t$ – the current time or the number of iterations.

- Z-score scaling.

As it is known^22^, Z-score scaling is a data normalization method that converts data into a standard scale with a mean of zero and a standard deviation of one. This allows comparing different data sets based on their distribution, which is useful for statistical analyses and machine learning models. Scaling helps to avoid problems associated with different units of measurement or ranges of values and improves the convergence of learning algorithms.

For the dataset under consideration in this paper (2), is used to apply Z-score scaling to numeric columns, such as CO_2_ concentration or air temperature.

2 $$\:Z=\frac{(X-\mu\:)}{\sigma\:},$$

where $\:Z$ – the normalized value for each record, $\:X$ – the value in the row (e.g., measured CO_2_), $\:\mu\:$ – the mean value for the entire column, and $\:\sigma\:$ is the standard deviation for the entire column.

2) ML models (linear regression, SVM polynomial, SVM rbf, Gradient boosting, Adaboost).

To use different machine learning models as surrogate models for the aforementioned dataset, we need to follow a few steps. First, we need to process the date and time columns and make sure that all data is in numeric format. We will also divide the dataset into training and testing. We will train and evaluate several models: Linear regression^23^, SVM with polynomial and RBF kernels^24,25^, Gradient Boosting^26^ and Adaboost^27^. In particular, such a simple model as linear regression is suitable for data that has a linear relationship. SVM with a polynomial kernel is used to detect non-linear dependencies in data. The polynomial kernel allows the model to create nonlinear solutions. SVM with RBF kernel helps in modeling complex, non-linear dependencies. This model is especially useful for complex distributions. Gradient Boosting is known as an ensemble technique that combines weak models, such as decision trees, to improve overall accuracy. Another ensemble approach is AdaBoost, which increases the weight of false predictions by emphasizing the correction of errors in previous models.

The effectiveness of each model is evaluated based on the Mean Square Error (MSE), The Root Mean Square Error (RMSE), and the Coefficient of Determination (R2). The MSE statistic measures the average square of the differences between the observed (actual) and modeled (predicted) values. MSE assesses how accurately the model reflects the real data, paying special attention to large deviations, which makes it sensitive to significant errors. The MSE is calculated using the (3).

3 $$\:MSE=\frac{1}{T}\sum\:_{i=1}^{T}{\left({O}_{i}-{S}_{i}\right)}^{2}$$

where $\:{O}_{i}$ – is the observed value, $\:{S}_{i}$ – is the simulated value, $\:T$ – is the total number of observations.

The Root Mean Square Error (RMSE) is a commonly employed statistical metric that quantifies the average magnitude of prediction errors between simulated (predicted) and observed (actual) values. It serves as a key measure for evaluating the accuracy of predictive models. RMSE computes the square root of the mean of the squared differences between predicted and observed values, thereby highlighting the impact of larger errors and providing a clear assessment of model performance. The RMSE is calculated using the (4).

4 $$\:RMSE=\sqrt{\frac{1}{T}\sum\:_{i=1}^{T}{\left({O}_{i}-S\right)}^{2}}$$

where $\:{O}_{i}$ – is the observed value, $\:{S}_{i}$ – is the simulated value, $\:T$ – is the total number of observations.

Mean Absolute Error (MAE) is a measure of the average magnitude of errors between simulated (predicted) and observed (actual) values. It gives an overall idea of how wrong the predictions are, on average, in the same units as the data itself. MAE is particularly useful when you want a simple representation of prediction error without considering the direction (overestimation or underestimation). The MAE is calculated using the (5).

5 $$\:MAE=\frac{1}{T}\sum\:_{i=1}^{T}\left|{O}_{i}-{S}_{i}\right|$$

where $\:{O}_{i}$ – is the observed value, $\:{S}_{i}$ – is the simulated value, $\:T$ – is the total number of observations, || – denotes the absolute value.

The R^2^ is a statistical measure that indicates how well the regression model approximates the real data points. It is a key metric used in regression analysis to evaluate the goodness-of-fit of the model. R^2^ represents the proportion of the variance in the dependent variable (y) that is predictable from the independent variables (x) in the regression model. In simpler terms, R^2^ explains how well the regression model predicts or explains the variability in the dependent variable. The R^2^ is calculated using the (6).

6 $$\:{R}^{2}=1-\frac{\sum\:_{i=1}^{T}\left({O}_{i}-{S}_{i}\right)}{\sum\:_{i=1}^{T}\left({O}_{i}-\stackrel{-}{{O}_{i}}\right)}$$

where $\:{O}_{i}$ – is the observed value, $\:{S}_{i}$ – is the simulated value, $\:T$ – is the total number of observations, $\:\stackrel{-}{{O}_{i}}=\frac{1}{T}\sum\:_{i=1}^{T}{O}_{i}$ – is the Mean of the observed values. Lower MSE values indicate better predictions, and R^2^ values closer to 1 indicate a better fit.

3) Long Short-Term Memory (LSTM) with Rolling Window Cross-Validation.

LSTM networks can be effectively used as surrogate models in various applications where capturing temporal dependencies is crucial. A surrogate model is a simplified representation of a more complex and computationally expensive model. The use of LSTMs as surrogate models is particularly beneficial in scenarios involving time-series data, sequences, or any system where the state evolves over time.

The proposed method integrates the power of LSTM networks with Rolling Window Cross-Validation (RWCV) to address the challenge of evaluating time-series models effectively. This combination allows for better capturing of temporal dependencies, particularly in scenarios where time-evolving data is critical. While LSTM is commonly used for time-series forecasting, incorporating RWCV introduces an innovative evaluation strategy that ensures model robustness and adaptability to dynamic changes in data across different time horizons.

**Fig. 1:**
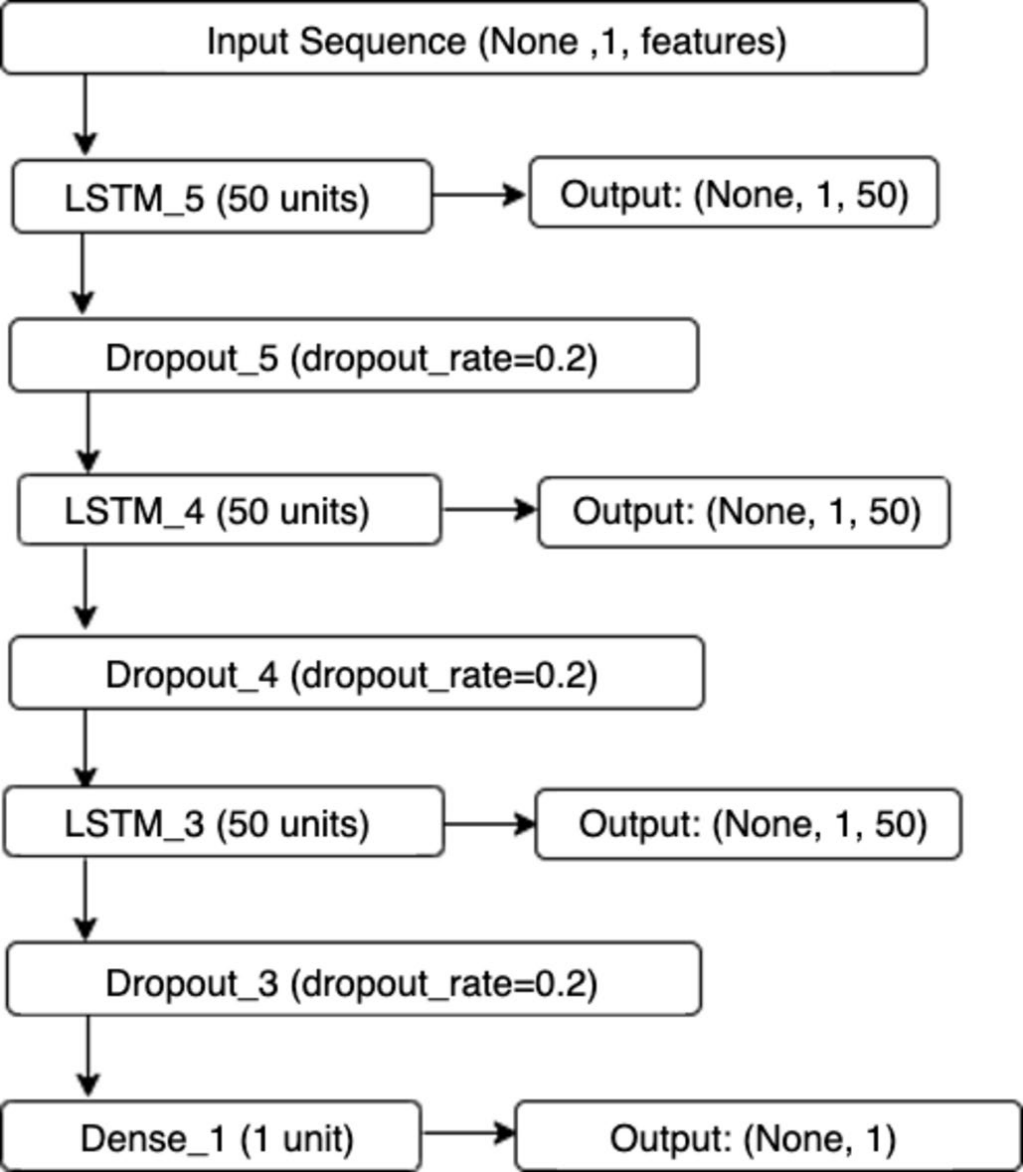
Flowchart visualization for an LSTM model.

Loss function is calculated as a mean squared error. Adam optimizer is implemented. Grid search was implemented for hyperparameters choosing. The proposed architecture of the LSTM model is shown in Fig. 1.

The LSTM layers are sequentially stacked with the specified number of units (50). The first LSTM layer outputs a sequence (None, 1, 50), which is passed to subsequent layers. Dropout Layers are included to prevent overfitting by randomly setting a fraction of input units to 0 at each update during training. Dense Layer is used to produce the final output, reducing the dimensionality to the desired output shape (None, 1). The total number of parameters is the sum of parameters from all layers, totaling 50,851. Thus, the amount of memory occupied by the proposed LSTM model, taking into account float32 for storing parameters, is approximately 203.4 kilobytes. The detailed breakdown helps in understanding the structure and the complexity of the model. 100 epochs are used for the training process, Batch size is equal to 1, dropout_rate = 0.2, learning_rate = 0.001.

Rolling Window Cross-Validation (RWCV) is a technique used to evaluate models on time series^28^. It involves gradually increasing the size of the training data set while maintaining the order of the time points. Here $\:y=\left\{{y}_{1},\:\:{y}_{2},\dots\:,\:{y}_{T}\right\}$ – a time sequence of $\:T$ observations, $\:k$ – the number of cross-validation splits (folds), $\:h$ – the forecasting horizon (the number of steps forward for the test set), and, $\:w$ – the size of the sliding window.

The data is divided into $\:k$ parts such that each partition has a training set and a test set. The training set is gradually increased and the test set moves forward by the same interval $\:h$.

Next, training and test sets are formed for each fold $\:i$:

- Training set: $$\:{y}_{train}^{i}=\left\{{y}_{1},\dots\:,{y}_{{t}_{i}}\right\}$$,
- Test set: $$\:{y}_{test}^{i}=\left\{{y}_{{t}_{i}+1},\dots\:,{y}_{{t}_{i}+h}\right\}$$,

where $\:{t}_{i}$ –the index of the training set for the $\:i$ -th fold, and $\:h$ is the number of points in the test set.

The training set is consistently increased by the following steps:

- In each subsequent fold $\:i$, the size of the training set is increased, while the test set remains fixed.
- Training set for fold $\:i$: $\:{y}_{train}^{i}=\left\{{y}_{1},\dots\:,{y}_{w+i*h}\right\}$.
- Test set for fold $\:i$: $\:{y}_{test}^{i}=\left\{{y}_{w+i*h+1},\dots\:,{y}_{w+(i+1)h}\right\}$.

Thus, in each fold, the training data window “slides” forward by $\:h$ points.

**Fig. 2:**
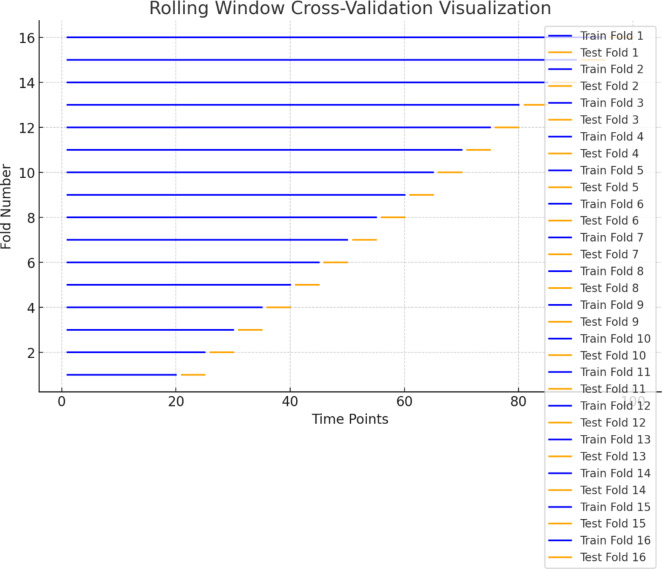
RWCV visualization for an LSTM model.

The visualization in Fig. 2 demonstrates how Rolling Window Cross-Validation (RWCV) works over a time-series dataset.

Blue lines (Fig. 2) represent the training sets for each fold, which gradually increase in size as the window slides forward. Orange lines represent the corresponding test sets, which always remain the same size (in this case, h = 5). For each fold, the training set grows, including all prior data points, and the test set slides forward by h time points. This method ensures that the model only sees past data during training and is evaluated on future, unseen data in each fold.

The evaluation framework incorporates RWCV, ensuring temporal integrity by maintaining the chronological order of data during training and testing. Metrics like MSE and R² are recalculated dynamically for each fold in the rolling window, providing accurate assessments aligned with evolving data patterns.

4) Time series (ARIMA).

The ARIMA (AutoRegressive Integrated Moving Average) model is a popular time series forecasting method. It is defined by three parameters: p, d, and q. These parameters are specified in the model as ARIMA(p, d, q).

AR (AutoRegressive) component (p) = 1 indicates that the model uses 1 lag of the dependent variable (the target variable) to make predictions. Essentially, it means that the current value of the series is based on the immediately preceding value (lag 1).

I (Integrated) component (d) = 0 indicates that the data does not need to be differenced to become stationary. Differencing is a technique used to make a time series stationary by subtracting the previous observation from the current observation^29^. A value of “0” means no differencing is applied.

MA (Moving Average) component (q) = 0 indicates that no lagged forecast errors are used in the model. This means that the model does not use past forecast errors to predict the current value.

5) Two time series comparison.

Comparing two time series can be done through visual inspection, statistical metrics, correlation analysis, cross-correlation, dynamic time warping, mean squared error, and statistical tests. The Augmented Dickey-Fuller (ADF) test is a common statistical test used to determine whether a time series is stationary or contains a unit root (i.e., it is non-stationary)^30^. Dickey-Fuller statistic for Measured_IAT is equal to -3.5895 i.e., the series is non-stationary. Lag order indicates the number of lagged difference terms included in the test to account for autocorrelation in the series. In this case, 13 lags were included. p-value is equal to 0.03367. This is the probability of obtaining a test statistic as extreme as, or more extreme than, the observed value under the null hypothesis. A lower p-value indicates stronger evidence against the null hypothesis. Augmented Dickey-Fuller Test for simulated value is the following: Dickey-Fuller = -3.7806, Lag order = 11, p-value = 0.01996, alternative hypothesis: stationary.

The KPSS (Kwiatkowski-Phillips-Schmidt-Shin) test is used to test for the stationarity of a time series. Specifically, it tests the null hypothesis that a time series is level or trend stationary against the alternative hypothesis that it is a unit root process (i.e., non-stationary). Measured_IAT: KPSS Level = 22.57, Truncation lag parameter = 8, p-value = 0.01.

## Results and discussion

Two different houses were analyzed during winter and summer. The first house has first and second-grade floor, while the second house has zero and first-grade floor. The error (the difference between the measured and predicted IAT value) can be either positive or negative from the exact value. Consequently, the cumulative error of the calculations can be reduced, i.e., be less than each of the true errors of the quantities (input data) involved in the calculations. The true cumulative error may even be zero.

We first conduct a visual analysis of the cumulative error for different rooms and in different seasons (see Fig. 2).

**Fig. 3:**
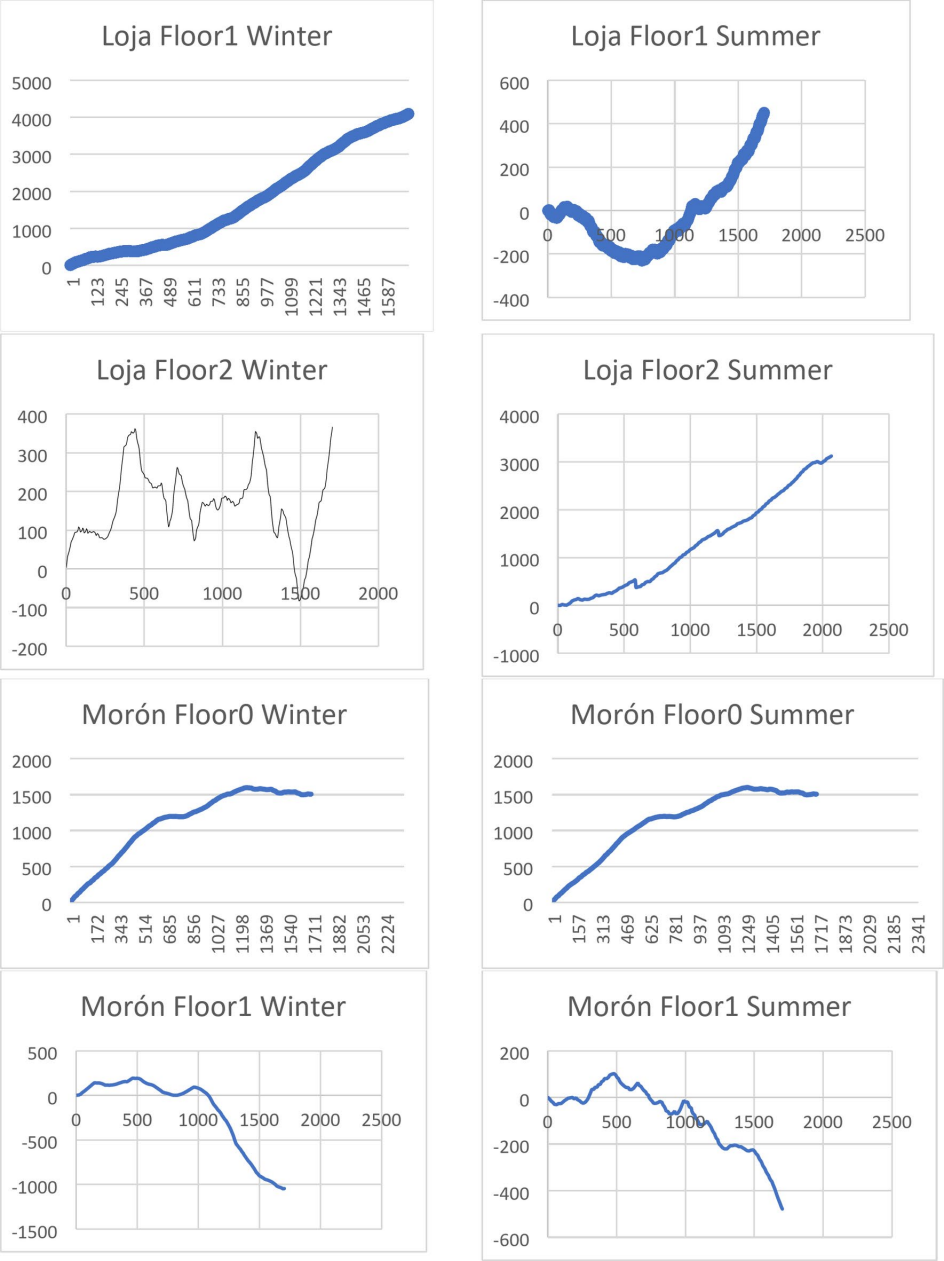
Visual representation of cumulative error for different rooms and seasons.

As shown in Fig. 3, the nature of the cumulative error function varies across all eight datasets and is independent of building type, season, and flooring.

Since cumulative errors represent a special case of the AR 1 model for ρ1 = 1, we start with a more general solution that is valid for any ρ1. Consequently, we used Adaboost, Gradient boosting, and linear regression in our analysis. The comparison of separated houses in different seasons is given in Table 2.

**Table 2:** Comparison of detached houses in different seasons using various prediction metrics.

Building	Loja				Moron			
Floor	1		2		0		1	
Season	Winter	Summer	Winter	Summer	Winter	Summer	Winter	Summer
R2								
Linnear regression	0.967	0.521	0.898	0.974	0.859	0.999	0.731	0.836
Adaboost	1	1	0.999	1	1	1	1	1
Gradient boosting	1	1	0.983	1	1	1	1	0.999
MSE								
Linnear regression	56015.59	13151.87	689.46	13478.40	30784.31	548.97	43992.63	2939.94
Adaboost	4.26	0.63	6.14	8.167	1.58	0.32	1.28	0.44
Gradient boosting	17.93	12.96	153.91	160.64	11.70	4.69	17.37	19.45
RMSE								
Linnear regression	236.676	114.682	75.821	116.096	175.455	23.431	209.744	54.221
Adaboost	2.065	0.791	2.478	2.858	1.256	0.569	1.132	0.662
Gradient boosting	4.234	3.601	12.406	12.675	3.421	2.166	4.168	4.411
MAE								
Linnear regression	205.042	2.753	82.411	91.182	154.232	17.588	183.163	44.143
Adaboost	1.044	0.412	1.279	1.554	0.597	0.288	0.595	0.303
Gradient boosting	3.217	91.181	9.207	6.421	2.629	1.648	3.229	3.393

As observed, the best model in terms of MAE, RMSE, MSE, and coefficient of determination errors was the Adaboost model. Next, the model was calibrated using LSTM, a process involving the adjustment of parameters and hyperparameters to achieve high prediction accuracy. LSTM is particularly well-suited for handling time series and sequential data. Hyperparameter selection was carried out using the Grid Search method.

**Fig. 4:**
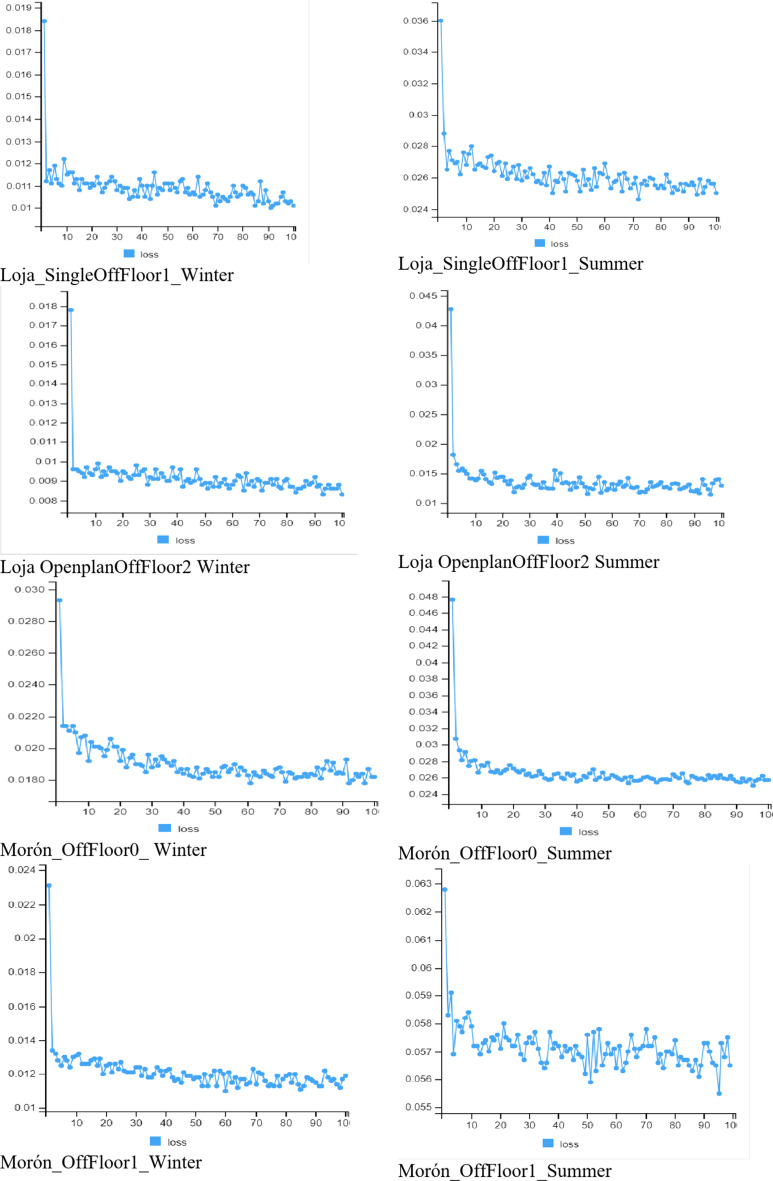
Loss or training dataset.

Cross-validation in the context of time series is a bit different from regular cross-validation, as the data is in a temporal sequence and this order needs to be preserved. The Rolling Window Cross-Validation approach was used, where a sliding window was used to split the data into training and test sets. After each step, the window is moved forward by several points. Figure 4 shows the test loss across the eight datasets.

Figure 4 shows that the model trained equally well on all eight datasets. Table 3 shows the loss values on the test data.

**Table 3:** Testing loss for LSTM and LSTM with WRCV.

Model	Loja				Moron			
	1		2		0		1	
	Winter	Summer	Winter	Summer	Winter	Summer	Winter	Summer
LSTM	0.009345	0.018235	0.01564	0.0006356	0.004642	0.04124	0.00332	0.01652
LSTM with WRCV	0.00827	0.01471	0.00956	0.0004709	0.00321	0.02819	0.00195	0.00931

The total testing loss varies from 0.00047 to 0.02819, depending on the specific season and floor. For example, the lowest losses are observed for the winter period on floor 1 in Loja – 0.00827 and floor 0 in Moron – 0.00195. In the summer, the losses are slightly higher, especially for Moron on floor 2–0.02819, but in general, the model demonstrates stable accuracy in predicting temperature conditions in different building operating conditions.

These results confirm that the proposed LSTM model effectively predicts temperature conditions in the Loja and Moron buildings with high accuracy, which is important for applications in energy-efficient technologies and indoor climate management.

In the standard setting of LSTM, temporal dependencies are captured but with a fixed dataset size. The model may only adapt well to new trends if the training dataset is representative of all possible variations in the data. The usual LSTM may need help with forecasting future values if trends in the data shift over time since the model isn’t continuously updated or retrained with newer data.

The proposed LSTM with RWCV incorporates dropout layers between LSTM units, which prevent overfitting by randomly disabling neurons during training, helping the model to generalize more effectively to unseen data. Since the model is trained on multiple subsets of data through RWCV, the risk of overfitting to any single partition of data is reduced. The model learns across multiple windows, further improving generalization. Although dropout layers can also be applied in standard LSTM architectures, the risk of overfitting remains higher because the model is often trained on a single, fixed dataset. Without the RWCV, a conventional LSTM may not generalize as effectively, especially in cases of non-stationary data where trends and patterns vary over time.

Furthermore, the proposed LSTM with RWCV is designed to be memory-efficient, with only 50,851 parameters, occupying approximately 203.4 kilobytes of memory using float32 precision. This makes it well-suited for applications where computational resources are limited. The grid search and rolling window validation add computational complexity but provide a more fine-tuned and robust model.

The rolling window cross-validation approach enhances the model’s ability to generalize to new, unseen data by simulating a real-time forecasting scenario. By continuously exposing the model to new data, it becomes more adaptive to evolving patterns in the time-series. In contrast, the usual LSTM model does not adapt as easily to new data unless it is explicitly retrained with a new dataset. Without the rolling window mechanism, the generalization capability is constrained by the original training data, which may not adequately capture future trends or shifts in the dataset.

To further establish the novelty and robustness of the proposed LSTM-based surrogate model, we conducted an additional comparative analysis with state-of-the-art techniques in time-series modeling. These include:

1. Transformers for time-series forecasting have emerged as a powerful tool for time-series analysis due to their self-attention mechanism, which captures both short- and long-term dependencies in the data. The integration of positional encoding allows Transformers to effectively model temporal patterns. In our experiments, we adapted the Transformer architecture with hyperparameters optimized for indoor air temperature prediction and evaluated its performance using the same metrics (MSE, RMSE, and R²).
2. Hybrid CNN-RNN models. Combining the feature extraction capabilities of Convolutional Neural Networks (CNNs) with the sequential learning strengths of Recurrent Neural Networks (RNNs), hybrid models offer a compelling approach for time-series forecasting. In this study, we implemented a CNN-LSTM hybrid model, where CNN layers processed spatial dependencies, and LSTM layers captured temporal dependencies.
3. Other state-of-the-art algorithms are also utilized. Bidirectional LSTM (BiLSTM) extends the LSTM by incorporating both forward and backward temporal dependencies, enhancing its ability to model sequential data. Temporal Convolutional Networks (TCNs) are convolutional architectures specifically designed for processing sequential data.
4. Classical ML approaches, were employed^22–26,31^. Adaboost and Gradient Boosting were chosen for their effectiveness in handling small datasets and their ability to model complex patterns through ensemble learning. The study did not include models like XGBoost or advanced neural networks due to their higher computational demands, aligning with the study’s focus on lightweight, computationally efficient models suitable for real-time applications.

The results of these comparisons are summarized in Table 4 and calculated for the entire dataset. While the Transformer model demonstrated strong performance on datasets with complex and irregular patterns, it required significantly higher computational resources compared to LSTM. The CNN-LSTM hybrid model exhibited improved performance in datasets with high spatial variability, but its training time was longer due to the added CNN layers.

Interestingly, the BiLSTM provided marginal gains in accuracy but at the cost of increased model complexity. TCNs, while computationally efficient, struggled with datasets exhibiting non-stationary trends, making them less suitable for dynamic environments.

**Table 4:** Comparative performance of state-of-the-art techniques for IAT prediction.

Model	MSE (↓)	RMSE (↓)	R2 (↑)	Training Time (↓)	Scalability	Notes
LSTM with RWCV	**0.0083**	**0.091**	**0.993**	**Low**	High	Best balance of accuracy, adaptability, and computational efficiency.
Transformer	0.0097	0.098	0.990	High	Moderate	Excels in long-term dependencies; computationally intensive.
CNN-LSTM	0.0102	0.101	0.987	Moderate	High	Strong in datasets with spatial dependencies; requires additional computational resources.
BiLSTM	0.0090	0.095	0.992	Moderate	Moderate	Slightly improved accuracy; increased complexity with bidirectional modeling.
TCN	0.0125	0.112	0.980	Very Low	High	Computationally efficient; struggles with non-stationary data trends.
Gradient Boosting	0.0143	0.120	0.972	Low	High	Reliable ensemble model; limited in handling temporal dependencies without feature engineering.
AdaBoost	0.0172	0.131	0.960	Low	High	Robust performance; less suited for non-linear time-series data.
Linear Regression	0.0241	0.155	0.920	Very Low	High	Simple and interpretable baseline; struggles with complex dependencies.
Lasso Regression	0.0221	0.148	0.925	Low	High	Effective for sparse datasets; reduced feature complexity.
Ridge Regression	0.0209	0.144	0.932	Low	High	Regularization improves stability but adds computation.

LSTM with RWCV emerges as the most practical solution, excelling in accuracy and computational efficiency for real-time applications. The LSTM with RWCV is more computationally efficient and scalable than traditional LSTMs due to its memory-efficient design with only 50,851 parameters occupying ~ 203.4 KB. It uses rolling window cross-validation (RWCV) to dynamically adjust the training set size, ensuring adaptability to new data trends in real-time. This mechanism reduces overfitting and enhances the model’s ability to generalize across diverse time-series datasets, addressing limitations of traditional LSTMs that rely on static datasets.

Transformer models deliver comparable accuracy but demand higher computational resources, making them less practical for certain intelligent building setups. Hybrid CNN-LSTM and BiLSTM models perform well but at the cost of increased complexity. TCNs, while computationally efficient, are less versatile in dynamic and non-stationary environments.

Table 4 highlights each approach’s comparative strengths and trade-offs, reinforcing the utility of the proposed LSTM-RWCV model.

## Conclusions

The findings of this study provide significant implications for improving indoor climate management, guiding policy development, and informing future research directions. These implications highlight the study’s potential to contribute to advancements in the design, operation, and management of energy-efficient buildings.

From a practical perspective, the LSTM with RWCV framework offers a highly accurate, scalable, and computationally efficient solution for predicting IAT. Its integration into building management systems enables dynamic optimization of HVAC operations, ensuring occupant comfort while reducing energy costs. The lightweight and resource-efficient architecture of the proposed model further enhances its suitability for deployment in resource-constrained settings, such as older buildings or infrastructure lacking advanced technological capabilities. By reducing energy consumption and optimizing HVAC operations, the framework supports sustainability efforts and mitigates greenhouse gas emissions. Additionally, its ability to adapt to dynamic and non-stationary data ensures robust performance in diverse operational scenarios, making it a valuable tool for large-scale implementations in smart cities where rapid adaptation to evolving conditions is essential.

The findings also carry significant implications for policy development. The predictive capabilities of the proposed framework can facilitate compliance with increasingly stringent energy efficiency and sustainability regulations. Policymakers can leverage such models to enforce building codes that promote greener infrastructure and energy conservation. Additionally, governments and institutions could introduce incentives, such as subsidies or tax reductions, to encourage the adoption of advanced machine learning models in building energy management systems. These incentives would promote investment in energy-efficient technologies and support broader sustainability goals. Moreover, as IoT and AI technologies become more prevalent in building management, it is crucial to establish robust frameworks that ensure data privacy and security, fostering trust and enabling widespread adoption of predictive models.

The study also opens up several opportunities for future research. One important direction is to test the model’s performance across diverse building types, geographic regions, and climatic conditions to assess its generalizability and applicability in varied contexts. Research could also explore scalability in more complex or multi-zone building environments, where diverse systems operate simultaneously. The development of hybrid modeling approaches, such as combining the LSTM-RWCV framework with physics-informed neural networks, could enhance interpretability and robustness, particularly in extreme or edge-case scenarios. Additionally, integrating predictive climate models with renewable energy systems, such as solar and wind power, could dynamically optimize energy use and improve the sustainability of building operations.

The LSTM network with RWCV is used for time series modeling, which allowed to significantly improve the consideration of time dependencies in the prediction of internal temperature. The use of LSTM as a surrogate model provides a novel approach to replacing more complex and computationally expensive models, particularly in time-sensitive applications. By reducing the need for intricate models, the proposed LSTM architecture provides faster predictions while maintaining high accuracy, making it an efficient surrogate solution in various time-series applications.

The loss values varied based on the building’s floor and the season. For instance, winter conditions on the ground floor exhibited the lowest loss values (e.g., 0.0004709), while summer conditions on higher floors showed higher losses (e.g., 0.02819861). These conditions were defined by factors such as seasonal indicators (e.g., temperature and humidity variations) and the specific floor being analyzed, which affected the indoor air temperature (IAT) dynamics.

The model is optimized for memory efficiency, with a total of 50,851 parameters requiring approximately 203.4 kilobytes when stored using float32. This compactness demonstrates the model’s practicality in environments with limited computational resources, setting it apart from more memory-intensive architectures. The integration of RWCV allowed the model to adapt dynamically to evolving data trends, effectively addressing non-stationary patterns in building environments. This aligns with the research goal of enabling real-time adaptability.

Further research should also address long-term error accumulation by investigating advanced error analysis mechanisms, such as cumulative error corrections or periodic model retraining. Incorporating occupant behavior and activity patterns as variables in predictive models could lead to more user-centric and efficient climate management solutions, improving energy optimization while ensuring comfort. Finally, the methodologies developed in this study have potential applications beyond building climate management. Extending the framework to other domains, such as transportation systems or industrial process optimization, could provide valuable insights for energy management and environmental control in other sectors.

In conclusion, this study provides a robust foundation for advancing indoor climate management in smart buildings by addressing critical gaps in predictive modeling. The findings offer practical solutions for enhancing energy efficiency, inform policies that incentivize sustainable practices, and identify future research directions to improve the generalizability, scalability, and adaptability of predictive models. Collectively, these contributions position the proposed framework as a key enabler of sustainable and intelligent building technologies, addressing the challenges of increasing environmental and economic pressures.

The study demonstrated practical applications in two buildings during winter and summer seasons, achieving high prediction accuracy with low loss values. The surrogate modeling approach showed potential for optimizing HVAC systems, reducing energy consumption, and maintaining comfortable indoor climates. These results support the model’s applicability in real-world smart building management.

Future research should focus on integrating advanced technologies (IoT, hybrid models, renewable energy systems) and addressing diverse building types, climates, and extreme scenarios. These directions will not only improve model performance but also promote sustainable energy usage and smarter building management. While the model demonstrated strong generalization capabilities, it faced challenges in scenarios with sparse or non-representative data, particularly in extreme climate conditions. Moreover, dataset size and diversity significantly impact model performance. The study emphasized that the model struggled with certain partitions due to insufficient data diversity. Future plans include expanding the dataset to cover a wider range of scenarios and increasing variability in building conditions.

## Data Availability

All data are fully available without restriction. They may be found at: https://data.mendeley.com/datasets/fj23wz552c/1.Caro, Rosana (2024), “MunicipalitiesAndalusiaSpain2019_SimulatedMonitored”, Mendeley Data, V1, doi: 10.17632/fj23wz552c.1.
